# The emerging role of pancreatic exocrine fibrosis as a common aetiological driver of islet dysfunction and diabetes: opportunities for novel disease-modifying interventions

**DOI:** 10.1007/s00125-026-06678-6

**Published:** 2026-02-10

**Authors:** Nicole Kattner, Ayat Bashir, James A. M. Shaw

**Affiliations:** 1 Translational and Clinical Research Institute, Newcastle University, Newcastle upon Tyne, UK; 2 Institute of Transplantation, Freeman Hospital, Newcastle upon Tyne Hospitals NHS Foundation Trust, Newcastle upon Tyne, UK

**Keywords:** Chronic pancreatitis, Cystic fibrosis-related diabetes, Fibrosis, Nintedanib, Pancreatic ductal adenocarcinoma, Pancreatic stellate cells, Pancreatogenic diabetes, Pirfenidone, Review, TGF-β, Type 3c diabetes

## Abstract

Three diseases primarily affecting the exocrine pancreas—chronic pancreatitis, cystic fibrosis and pancreatic ductal adenocarcinoma—are all associated with a high incidence of diabetes. Together, they may account for more cases of diabetes than autoimmune type 1 diabetes. All forms of pancreatogenic (type 3c) diabetes are characterised by impaired insulin secretion but maintenance of significant islet beta cell mass, even in the presence of virtually complete destruction of the exocrine component of the gland. Pancreatic ductal injury and associated fibrosis are common features in chronic pancreatitis, cystic fibrosis and ductal adenocarcinoma. Increased peri-ductal fibrosis is also seen in type 2 and type 1 diabetes. Here, we review the literature regarding a potential common aetiological role of pancreatic fibrosis in the pathogenesis of type 3c, type 2 and type 1 diabetes. A vicious profibrotic signalling cycle involving injured ducts, pancreatic stellate cells and macrophages with increased levels of pancreatic tissue TGF-β at the core has increasingly been recognised as an essential driver of pancreatic exocrine fibrosis. We propose a second diabetogenic perpetual cycle comprising paracrine signalling between activated pancreatic stellate cells, macrophages and the islets themselves, leading to potentially reversible beta cell failure. The antifibrotic agents pirfenidone and nintedanib, thought to work primarily through suppression of TGF-β function, are used routinely in clinical practice for non-pancreatic indications, with a first trial in pancreatitis underway. Trials evaluating these licensed therapeutics that include primary diabetes-related endpoints and measures aiming to elucidate the underlying mechanisms of action merit consideration in type 3c diabetes and ultimately in type 2 diabetes and in combinatorial regimens in type 1 diabetes.

**Graphical Abstract:**

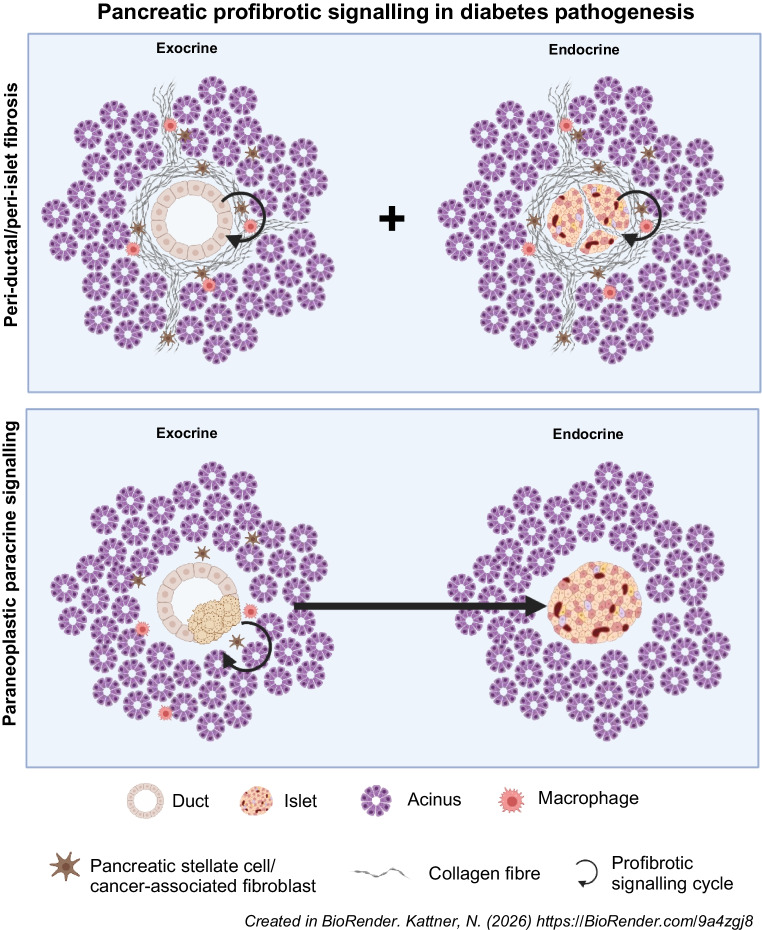

**Supplementary Information:**

The online version contains a slideset of the figures for download available at 10.1007/s00125-026-06678-6.

## Introduction

Relative or absolute deficiency of physiological insulin secretion is pathognomonic of diabetes mellitus, regardless of subtype or aetiology [1]. Mechanistic understanding of the processes in the human pancreas leading to impaired function and loss of beta cells remains remarkably limited. Circulating markers of the underlying disease processes have been largely limited to glucose and the islet hormones themselves, providing no insights into the underlying pathogenesis, with clinical imaging lacking islet-level resolution and an absence of biopsy pathology in living donors. However, having traditionally been considered an endocrine disease alone, pathology within the pancreatic exocrine gland has increasingly been recognised.

In this review we explore exocrine pancreatic cellular fibrosis and profibrotic signalling as a potential common pathway in the pathogenesis of beta cell insufficiency in diabetes across the spectrum of aetiologies, with a particular focus on diabetes secondary to chronic pancreatitis. We present evidence for a key coordinating role of TGF-β in a paracrine signalling cycle involving pancreatic stellate cells (PSCs), tissue-resident macrophages and pancreatic endocrine cells, leading to impaired insulin secretion. Finally, we summarise the potential of clinical trials using licensed antifibrotic agents with the goal of restoring normal beta cell function and glucose homeostasis in pancreatic pathologies associated with fibrosis and diabetes risk.

## Diabetes secondary to chronic pancreatitis

### Clinical phenotype

Chronic pancreatitis is a fibroinflammatory disease that occurs in individuals with underlying environmental, metabolic, genetic and/or other risk factors. The known prevalence is 12.6/100,000 people [2] but this is believed to be a significant underestimate as the condition often remains undiagnosed. It is becoming more common with the incidence doubling since 2015 [3]. Chronic pancreatitis is more common in men than women regardless of underlying aetiology [4]. Excess alcohol consumption is the commonest cause, with smoking being an additional risk factor. Hereditary pancreatitis is associated with mutations in genes including *PRSS1*, *SPINK1* and *CTRC* [5]. Autoimmune pancreatitis is part of a multiorgan IgG4-related disorder, usually presenting over the age of 60 years [6].

There is considerable heterogeneity in the clinical manifestation of chronic pancreatitis. Pain is a prominent symptom and is present at some point during the clinical course in 84–90% of individuals in longitudinal studies [7]. Exocrine digestive enzyme insufficiency affects approximately 70% of those with chronic pancreatitis of 20 years’ duration [8]. It is caused by obstruction of pancreatic outflow (e.g. pancreatic duct strictures or stones) or by loss of or damage to acinar cells. Chronic pancreatitis is the most common cause of pancreatogenic type 3c diabetes, with 30–40% of individuals affected after diagnosis of chronic pancreatitis [9] and up to 80% affected with long-standing pancreatitis [10]. True prevalence is likely to be higher given the frequent misdiagnoses, usually as type 2 diabetes. In a large retrospective study of UK primary healthcare records, type 3c diabetes was more prevalent than type 1 diabetes, affecting 1.8% vs 1.1% of the studied population [11]. Classically, a definitive diagnosis of chronic pancreatitis is dependent on the presence of at least intermittent bouts of significant abdominal pain in parallel with confirmation of abnormal pancreatic imaging including exocrine ductal abnormalities. However, type 3c diabetes may be associated with a less overt clinical syndrome; this emphasises the importance of taking a careful clinical history to identify possible previous pancreatitic episodes and concomitant pancreatic exocrine insufficiency in individuals presenting with diabetes with a phenotype characterised more by insulin insufficiency than by insulin resistance in parallel with visceral adiposity and other metabolic syndrome features.

Although insulin resistance associated with obesity, dyslipidaemia and family history of type 2 diabetes may play an exacerbating role [12], chronic pancreatitis-associated diabetes is characterised by insufficient pancreatic insulin secretion [13]. A higher AUC glucose response to a mixed-meal tolerance test associated with delayed time to peak C-peptide secretion has been reported in relatively short-duration chronic pancreatitis without diabetes [14]. Alpha cell dysfunction is also seen with initial aberrant glucose-induced hyperglucagonaemia, mirroring decreasing beta cell function [15], and a blunted response to hypoglycaemia [15, 16]. Pancreatic polypeptide secretion is reduced early in individuals with endocrine dysfunction associated with exocrine pancreatic disease and it has been proposed that this pancreatic polypeptide insufficiency mediates increased hepatic insulin resistance in chronic pancreatitis [17, 18].

### Pancreatic pathology

**Fig. 1:**
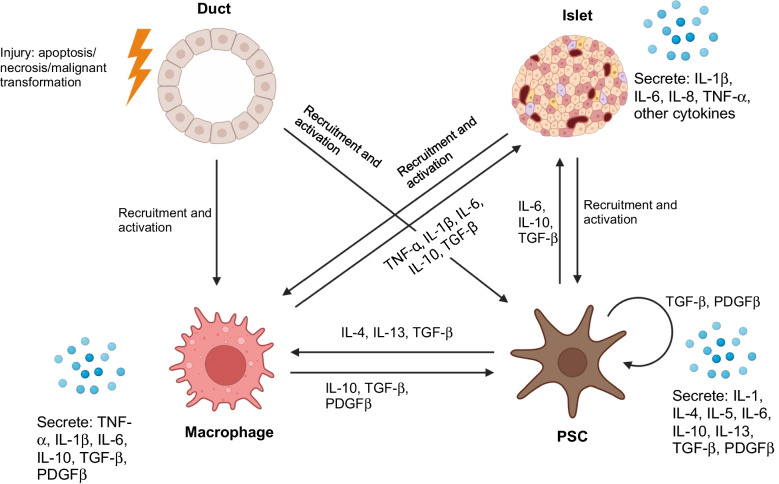
Schematic illustrating proposed exocrine and endocrine pancreatic profibrotic signalling networks. Ductal injury evidenced by apoptosis, necrosis or malignant transformation in pancreatic ducts leads to recruitment and activation of both macrophages and PSCs. Paracrine signals secreted by macrophages impact both PSCs and islets. PSC cytokine secretion impacts macrophages and islets in addition to having an autocrine effect that maintains the activated status. Islet endocrine cell cytokine signalling can recruit and activate macrophages and PSCs. Created in BioRender. Kattner, N. (2025) https://BioRender.com/wrkezym. This figure is available as part of a downloadable slideset

Pancreatic exocrine fibrosis, initially between lobules and surrounding ducts and then replacing areas of intralobular acinar parenchyma in parallel with pancreatic ductal pathology, is ubiquitously present in chronic pancreatitis of all aetiologies [19–22]. Fibrosis is associated with activated PSCs, characterised by alpha-smooth muscle actin (α-SMA) expression. PSCs are present in acinar lobules and regions of established fibrosis together with macrophages, and crosstalk between PSCs and macrophages has been reported previously [19, 21, 23–25]. Factors secreted by activated PSCs include IL-1, IL-4, IL-5, IL-6, IL-10, IL-13, TGF-β and platelet-derived growth factor (PDGF), which has been shown to promote macrophage polarisation towards an M2 (profibrotic) phenotype in in vitro experiments with primary cells [21, 23–25]. Activated PSCs produce and deposit collagen and other extracellular matrix components in the progressive fibrotic process in chronic pancreatitis [19, 26, 27]. It has been proposed that conversion from tissue repair to scarring fibrosis in chronic pancreatitis may be mediated by a perpetual cycle involving activated PSCs, macrophages and pancreatic duct cells [27–29] (Fig. 1).

Intact islets can be seen embedded within a fibrotic niche, even in the presence of end-stage exocrine pancreas pathology [20]. The overall pancreatic volume is decreased, with islets appearing relatively sparse but enlarged in size, particularly in advanced chronic pancreatitis [20, 30]. A decreased overall beta cell area (and thus proposed overall beta cell mass) has been observed, with 0.69±0.08% of the pancreatic section area comprising beta cells in chronic pancreatitis compared with 0.97±0.08% in control pancreases [20, 31, 32]. This is most pronounced in islets surrounded by fibrosis [31, 32], with the proportion of the pancreas comprising beta cells decreasing with worsening glucose tolerance [20, 33] and being lowest in chronic pancreatitis with established diabetes (0.41±0.09%) [20, 32]. Beta cell proliferation is rarely seen, with a rate comparable to that in age-matched control individuals [20]. Although beta cell apoptosis has been reported, this is not significantly greater than in control individuals [20]. Increased circulating proinsulin levels have been reported in chronic pancreatitis, suggesting beta cell dysfunction in parallel with absolute beta cell loss [34].

The overall alpha cell area (mass) in individuals with chronic pancreatitis appears comparable to that in control individuals (0.41±0.07% of total pancreatic tissue area in chronic pancreatitis vs 0.49±0.08% in control) [20] but an increase in the proportion of individual islets comprising alpha cells has been reported, particularly in islets surrounded by fibrosis [31]. The highest islet alpha cell proportion was seen in chronic pancreatitis donors with diabetes [32].

A potential increase in the proportion of somatostatin cells in chronic pancreatitis, especially in those with diabetes, was observed in a single study [32]. The observed proportion of pancreatic polypeptide cells varied widely between islets but appeared significantly higher in islets surrounded by fibrosis, despite decreased circulating pancreatic polypeptide levels [31].

**Fig. 2:**
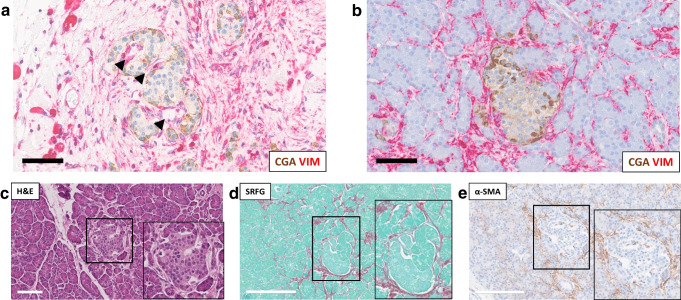
Histological and immunohistochemical assessment of an individual with chronic pancreatitis and a neuroendocrine tumour with duct obstruction. (**a**, **b**) Immunohistological staining for chromogranin A (brown; CGA) and vimentin (red; VIM). Staining highlights stromal tissue in widened vascular tracts (arrows in **a**) that is potentially separating islet endocrine cells in an islet surrounded by fibrosis (**a**) compared with an islet in a less fibrotic area of the tissue (**b**). H&E (**c**), SRFG (**d**) and α-SMA (**e**) staining of a single islet showing co-localisation of fibrosis and α-SMA expression. Further staining will be important to definitively distinguish activated PSCs (glial fibrillary acidic protein [GFAP]) from capillary pericytes (nerve/glial antigen 2 [NG2]). Tissue from the body (**a**) and head (**b**, **c**, **d**, **e**) region of a single resected pancreas. Scale bars: 70 µm (**a**, **b**), 100 µm (**c**), 200 µm (**d**, **e**). Inset panels (**c**, **d**, **e**) show higher magnification images of the boxed islet in the main photomicrograph. (**a**, **b**, **c**, **e**) Staining was performed by Novopath at the Royal Victoria Infirmary, Newcastle upon Tyne, UK. (**d**) Staining of SRFG was performed by Y. Al-Selwi in the Shaw laboratory (Newcastle University, Newcastle upon Tyne, UK). This figure is available as part of a downloadable slideset

Islets within fibrotic areas of the pancreas are typically clustered within the dense connective tissue, often with marked fibrosis around the microvasculature enlarging the peri-sinusoidal space [31]. Collagen fibre bundles may also divide islets into separate lobules, separating endocrine cells from the vasculature [31] (Fig. 2a). A murine chronic pancreatitis model has revealed changes to vascular morphology in parallel with fibrosis, with endothelial cell vacuolisation and irregular blood vessel surfaces at the ultrastructural level [30]. Despite alterations in endocrine cell ratios in affected islets, electron microscopy did not reveal degenerative lesions within beta cells, although insulin granule numbers appeared reduced. Fibrotic disruption of endocrine cell vascularisation and cell-to-cell connectivity or vascular pathology not associated with fibrosis may be indirect mediators of beta cell dysfunction and ultimately diabetes in chronic pancreatitis (Fig. 2a).

### Potential role of profibrotic signalling in islet endocrine dysfunction

**Fig. 3:**
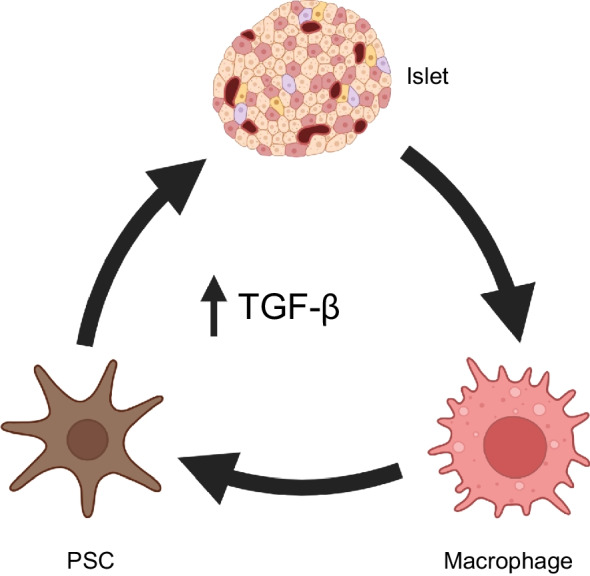
Increased tissue TGF-β expression has been shown in chronic pancreatitis [36] and in cystic fibrosis (Y. Al-Selwi, N. Kattner, J.A.M. Shaw, unpublished data). In the presence of elevated tissue TGF-β levels, a vicious profibrotic signalling cycle (indicated by arrows) is proposed involving activated macrophages, activated PSCs and ‘stressed’ dysfunctional islet endocrine cells. Created in BioRender. Kattner, N. (2026) https://BioRender.com/q0r8uhj. This figure is available as part of a downloadable slideset

An active cellular component of peri-islet fibrosis in chronic pancreatitis is confirmed by α-SMA staining for PSCs (Fig. 2c–e). Distance mapping has revealed close proximity of macrophages to activated PSCs around islets in chronic pancreatitis (B. Hunter, N. Kattner, J.A.M. Shaw, unpublished data). Profibrotic paracrine signalling may also play an important role in endocrine dysfunction in intact islets. Chronic pancreatitis is associated with high levels of inflammatory cytokines within the tissue including TGF-β, TNF-α, IFN-γ and IL-10 [35, 36]. Expression of IL-1β, IL-6, IL-8 and TNF-α by ‘stressed’ islet endocrine cells has been reported and we have identified beta cell IFN-γ expression in chronic pancreatitis (B Hunter, N Kattner, JAM Shaw, unpublished data). This may lead to JAK/STAT pathway activation [37], potentially decreasing expression of key transcription factors such as PDX-1 and thereby reducing insulin gene transcription. An altered endocrine cell phenotype, including a proportion of beta cells staining negative for the MafA transcription factor and urocortin-3 differentiation marker, has been reported in individuals with chronic pancreatitis without diabetes undergoing total pancreatectomy and islet autotransplantation [38]. In parallel with reduced beta cell PDX-1 staining in situ, impaired glucose-stimulated insulin secretion has been demonstrated in islets isolated from individuals with advanced chronic pancreatitis undergoing surgical resection [39]. From these circumstantial data showing M2 macrophages, activated PSCs and endocrine cells expressing proinflammatory cytokines in parallel with loss of an end-differentiated phenotype [40, 41], we propose a perpetual profibrotic signalling cycle within the islet niche in chronic pancreatitis in the presence of elevated tissue TGF-β levels that may drive islet dysfunction and ultimately diabetes (Fig. 3).

## Cystic fibrosis-related diabetes

Cystic fibrosis is an autosomal recessive genetic disorder leading to abnormal function of the cystic fibrosis transmembrane conductance regulator (CFTR), resulting in damaging viscous secretions in many organs. While primarily characterised by respiratory infection, pancreatic pathology is a ubiquitous early manifestation, with approximately 85% of affected children having pancreatic exocrine deficiency requiring enzyme replacement therapy [42]. Cystic fibrosis-related diabetes (CFRD) is the most common comorbidity and is associated with more frequent and severe pulmonary exacerbations and worse lung function. Progressive loss of beta cell function occurs in those with pancreatic exocrine insufficiency, with fewer than 10% of those with cystic fibrosis developing CFRD before the age of 10 years compared with a prevalence of 20% in adolescents, 50% in adults and up to 80% in those aged over 50 years with the most severe *CFTR* genotype [43–47]. The highest *CFTR* gene and protein expression within the pancreas is seen in ductal cells [48] and cystic fibrosis is associated with ductal occlusion with viscous secretions and dilatation in early life [49]. Through examination of post-mortem pancreases from donors over a relatively wide age range, we and others have concluded that exocrine pancreatic pathology in cystic fibrosis progresses from an early ‘fibrosis alone’ stage, with fibrosis largely confined to interlobular regions, to an advanced ‘fibrosis/liposis’ phase characterised by extensive peri-ductal fibrosis around distinctly dilated ducts, with virtually complete loss of acinar tissue and substantial areas of adipocyte replacement [50–52]. The ‘end-stage’ pancreas is atrophic with the majority of the gland comprising mature adipose tissue but with maintenance of densely clustered islets.

**Fig. 4:**
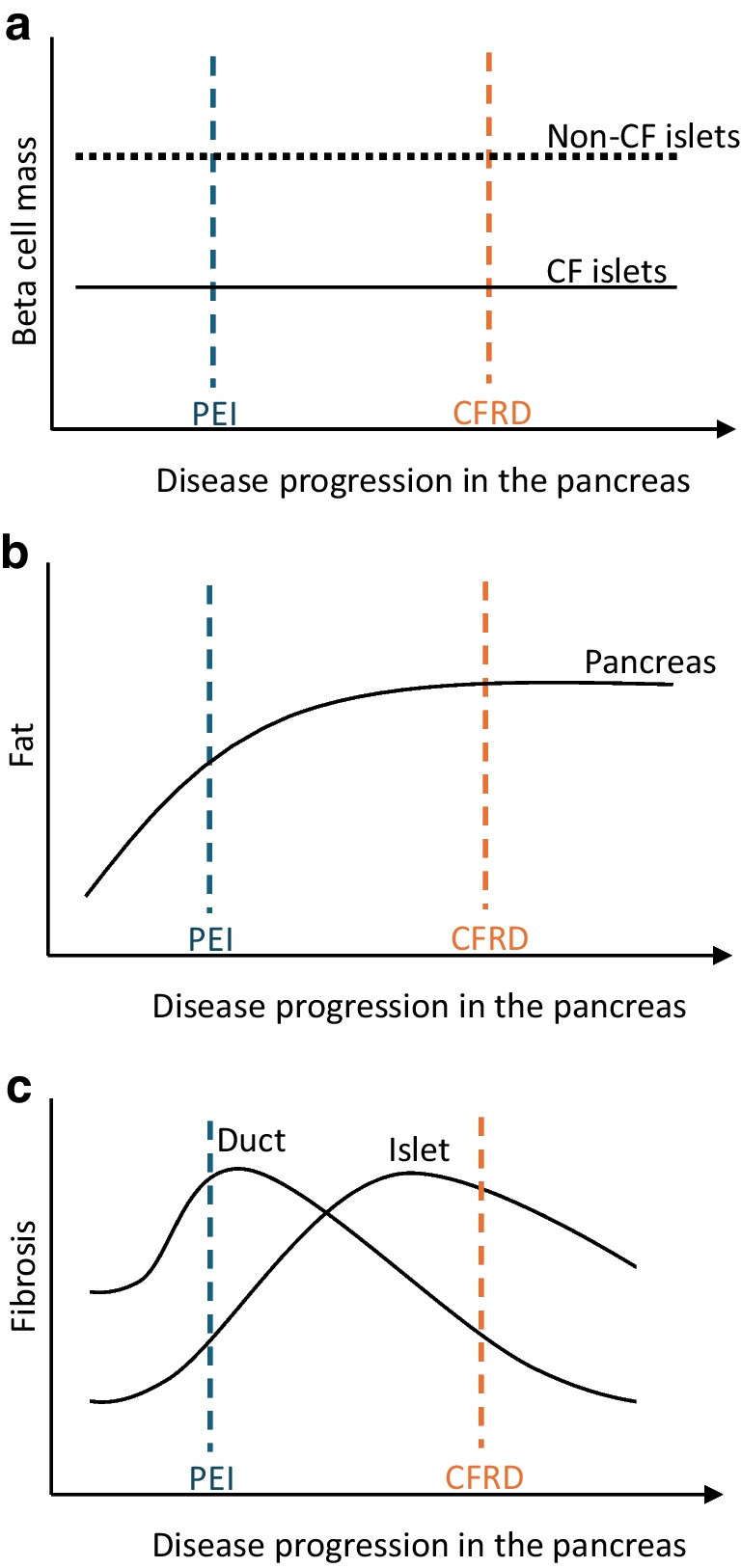
Schematic of beta cell mass (**a**), adipocyte replacement (**b**) and fibrosis in the microenvironments of ducts and islets (**c**) in relation to functional disease progression of cystic fibrosis within the pancreas to pancreatic exocrine insufficiency (PEI) and diabetes. (**a**) Beta cell mass is lower in cystic fibrosis than non-cystic fibrosis islets from birth with no further substantial reductions with onset of PEI or CFRD. This indicates that worsening glucose tolerance with increasing age is not primarily mediated by progressive loss of beta cell mass. (**b**) Pancreatic adipocyte proportional area increases rapidly during early postnatal life in parallel with loss of acinar mass and development of PEI. Virtually complete replacement of the gland by fat predates progression to CFRD, suggesting that liposis is not a primary driver of beta cell failure. (**c**) Fibrosis around pancreatic ducts increases early in the disease course of cystic fibrosis in tandem with development of PEI. Peri-ductal fibrosis then diminishes in parallel with ductal loss and replacement of acinar tissue by adipocytes. A second wave of fibrosis around and within islets appears to be temporally associated with progressive impairment in islet endocrine function [52]. This figure is available as part of a downloadable slideset

Systematic quantitative comparison of cystic fibrosis post-mortem pancreases with pancreases from control donors without pancreatitis or diabetes has confirmed no significant reduction in islet mass but progressive remodelling with decreased islet circularity [52]. Interestingly, the islet beta cell proportional area was reduced by almost 50% even in individuals with cystic fibrosis dying perinatally, suggesting the need for further investigation into the potential role of CFTR expression in normal islet development. Reduced beta cell proportional area is mirrored by an increase in the proportion of islets comprising alpha cells [52, 53]. There is no convincing evidence for further loss of beta cell mass with worsening glucose tolerance and progression to diabetes in cystic fibrosis, in keeping with a primary role of beta cell dysfunction as opposed to absolute loss of beta cells in CFRD pathogenesis. In our recent pseudo-timeline analysis [52], we concluded that decrease in beta cell mass (Fig. 4a) and global replacement of the exocrine pancreas with adipocytes (Fig. 4b) occur too early in the progressive pancreatic pathology to be the primary driver of CFRD.

### Potential role of profibrotic signalling in islet endocrine dysfunction

Peri-islet fibrosis, in addition to intra-islet collagen expanding the islet perivascular region and potentially impacting endocrine vascularisation and cell-to-cell connectivity, are present in advanced and end-stage cystic fibrosis [50, 52]. An active cellular component is evidenced by the presence of activated PSCs and macrophages in and around islets [52]. As has been proposed in chronic pancreatitis, we hypothesise that the presence of ‘stressed’ epithelial ducts as a result of CFTR mutation-associated dysfunction and dilatation is necessary to maintain a vicious cycle of fibrosis in cystic fibrosis. This is supported by resolution of exocrine fibrosis associated with ductal involution in advanced cystic fibrosis. Further, we postulate that there is a ‘second wave’ of fibrosis (Fig. 4c) centred on and actively involving signalling within the ‘stressed’ endocrine compartment in conjunction with activated PSCs and macrophages. This fibrotic process persists when the majority of the exocrine parenchyma has been replaced by fat. We propose that this second fibrotic cycle leads to CFRD through a paracrine effect that alters the islet cell phenotype and function (Fig. 3) and/or through a mechanical effect on vascularisation and endocrine cell communication (as exemplified in chronic pancreatitis; Fig. 2) in disorganised islets.

While the recent widespread availability of highly effective CFTR modulator therapy in cystic fibrosis has transformed respiratory outcomes and overall well-being, it is increasingly being concluded that these agents will not sustainably prevent progression to CFRD or improve glucose levels in established diabetes when commenced in individuals over the age of 12 years [54]. This is in keeping with the primary impact of *CFTR* mutation on pancreatic development and subsequent ductal pathology and the need for therapies beyond CFTR modulation. Evidence for islet-centric ongoing fibrosis following global exocrine pancreas replacement with fat suggests the possibility of novel therapeutic approaches targeting non-CFTR-mediated profibrotic signalling with the goal of preventing progressive pancreatic endocrine dysfunction.

## Diabetes in association with pancreatic adenocarcinoma

Pancreatic ductal adenocarcinoma (PDAC) is a leading cause of cancer-related mortality [55]. It affects more than half a million individuals worldwide with the prevalence predicted to rise by 70% by 2040 [55]. Risk factors include smoking, alcohol consumption, obesity and diabetes [55]. In a large prospective study, impaired fasting glucose was present in approximately 38% of people at the point of pancreatic cancer diagnosis and diabetes was present in 47% [56]. PDAC risk has been associated with duration of diabetes, with a higher risk (RR 5.38) in new-onset diabetes (<1 year) and a lower risk (RR 1.49) with a longer duration of diabetes (5–9 years) [57]. In a UK Biobank study, PDAC diagnosis was associated with worsening of glucose levels in established diabetes [58]. The relationship between new-onset diabetes and PDAC is striking, with diabetes detected a median of 10 months before cancer diagnosis [59]. An eightfold increased risk of PDAC in older individuals with new-onset diabetes compared with the non-diabetic population has been proposed, suggesting a common aetiology [60]. Diabetes cannot be explained by cancer cell invasion into islets; this has been observed in pancreatic biopsies but was not related to diabetes status [61]. Large clinical studies have shown that those with diabetes have larger tumours [62], but diabetes does not appear to be caused by physical destruction of a large proportion of islet mass, being present in 33% of individuals with a PDAC tumour diameter <10 mm [63]. New-onset diabetes is thus believed to be a paraneoplastic phenomenon where one or more tumour-secreted factors interfere with insulin secretion [64]. Metabolic assessment in PDAC has been undertaken in several small studies with one study reporting reduced beta cell function and increased insulin resistance in participants with PDAC compared with normoglycaemic control participants [65] and another reporting reduced insulin secretion without insulin resistance in participants with PDAC compared with those with type 2 diabetes [66]. An in vitro murine study reported induction of insulin resistance in a muscle cell line by pancreatic tumour-derived exosomes, supporting the presence of paracrine signalling beyond the pancreas [67]. Most recently, an in-depth comparison of the response to a mixed-meal tolerance test between 28 individuals with PDAC and 97 participants with type 2 diabetes, all with diabetes diagnosed within the preceding 3 years, robustly showed higher insulin sensitivity but lower insulin secretion (and higher post-meal glucagon secretion) in the PDAC group [68].

Insulin resistance, potentially in association with visceral adiposity, has been shown in epidemiological studies to be a risk factor for PDAC [69]. Using a mouse model, one study showed a detrimental impact of obesity-induced changes in islets on PDAC progression [70]. A decreased risk of pancreatic cancer has also been reported following bariatric surgery [71]. Existing evidence, however, supports impairment of islet function as opposed to induction of insulin resistance as the primary downstream paraneoplastic impact of pancreatic malignancy [69].

In a study of 104 individuals who underwent PDAC resection, 57% with new-onset diabetes had resolution of their diabetes postoperatively, supporting a paraneoplastic mechanism enabling recovery of normal glucose homeostasis even after surgical reduction of overall pancreatic endocrine mass [56].

Proposed factors secreted by PDACs as potential mediators of beta cell dysfunction include cytokines, hormones, metabolites and extracellular vesicles [72]. Decreased islet size and density have been reported in PDAC without evidence of altered morphology [73, 74]. Whether PDAC has a consistent impact on beta cell and alpha cell mass remains unclear. Both decreased (0.8±0.5% PDAC vs 1.13±0.6% control) and maintained (1.53±1.26% PDAC vs 0.95±0.42% control) beta cell area as a proportion of overall pancreatic area and an increased/unaffected beta cell:alpha cell ratio have been reported in separate post-mortem studies [31, 73–75].

### Potential role of profibrotic signalling in islet endocrine dysfunction

A role for pancreatic fibrosis in the development of PDAC is supported by the increased risk of pancreatic cancer in individuals with chronic pancreatitis vs non-chronic pancreatitis populations (RR 7.6–68.1 dependent on underlying aetiology) [76–79]. It is proposed that obesity increases PDAC incidence through pancreatic inflammation. Both PSC activation and extensive pancreatic fibrosis were seen in parallel with tumour formation in response to high-fat feeding in a mouse model of PDAC [80]. Pancreatic cancer is typically characterised by a relatively localised tumour surrounded by dense fibrotic stroma. This is produced by cancer-associated fibroblasts, which share many characteristics with activated PSCs, including α-SMA expression and cytokine secretion [81, 82]. These cytokines are believed to activate tumour-associated macrophages and mediate epithelial-to-mesenchymal transition in carcinoma cells, enhancing proliferation and migration [81, 83]. This is in keeping with our overarching model of ‘stressed’ (through malignant transformation in PDAC) ductal cells in a perpetual cycle with activated fibroblasts and macrophages, sustaining the loss of a differentiated epithelial phenotype. Through our proposed common mechanism linking profibrotic signalling to diabetes across a range of aetiologies, it can be postulated that paracrine signalling arising from the localised PDAC niche leads to cytokine-mediated global pancreatic endocrine cell dysfunction and diabetes (Fig. 1).

In addition to paracrine signalling to distant islets from a relatively small PDAC-associated profibrotic niche, in some cases extensive peri-ductal fibrosis may lead to widespread islet fibrosis and diabetes following ductal occlusion by a pancreatic head PDAC and distal dilatation. This has been described in six individuals, with comparable islet pathology to that of chronic pancreatitis, including a decreased beta cell:alpha cell ratio [31].

Cross-sectional histopathological studies can only show associations rather than prove causality and the mechanisms linking PDAC with obesity, insulin resistance, profibrotic signalling and diabetes remain unclear. It is hoped that ongoing clinical studies in the USA and UK will provide new insights into the early prediction and detection of PDAC in those with new-onset diabetes [84, 85].

## Potential role of fibrosis in type 2 diabetes pathogenesis

Development of type 2 diabetes in predisposed individuals is dependent on at least the relative failure of pancreatic insulin secretion. While this is associated with a decrease in beta cell area [86–90], this is not thought sufficient to fully account for the loss of physiological insulin secretory dynamics, with a parallel role proposed for dysfunction and loss of an end-differentiated phenotype [90–93]. Increasing pancreatic adipocytosis with increasing BMI has been consistently confirmed in imaging and histology studies [94, 95], with the potential for the highest levels in those with diabetes in a cohort of individuals with BMI >25 kg/m^2^ [96].

Type 2 diabetes is also associated with an increase in pancreatic fibrosis. Excess collagen distribution has been described as being primarily intralobular, but quantitative image analysis supports increased fibrosis in all regions including peri-/intra-islet [96–98].

A subgroup of deceased organ donors with known type 2 diabetes and no evidence of chronic pancreatitis had fibrosis affecting >40% of the pancreatic tissue area, which was potentially associated with a reduced islet beta cell:alpha cell ratio and the need for insulin treatment [96]. A previous systematic review concluded that type 2 diabetes was associated with specific exocrine changes including increased fibrosis and acinar atrophy, but no pathological and clinical features of pancreatitis such as ductal changes, significant inflammation and pain [99]. These findings support the possibility of a fibrotic phenotypic subtype of type 2 diabetes [100, 101] with more severe beta cell insufficiency than in individuals with predominantly adipocytic pancreatic exocrine pathology. This subtype may benefit from therapeutics targeting profibrotic signalling pathways rather than interventions addressing adiposity and insulin resistance alone.

In contrast to chronic pancreatitis and cystic fibrosis, in which fibrosis is clearly associated with pathological changes primarily affecting the exocrine pancreas, it remains possible that fibrosis in type 2 diabetes is secondary to primary changes within the endocrine compartment, such as macrophage recruitment and activation in response to ‘stressed’ beta cells in the presence of hyperglycaemia, with parallel elevated islet insulin secretion in the earlier stages of disease progression [41, 102]. Cytokines secreted by macrophages within the islet niche in type 2 diabetes, including TNF-α, IL-1β, IL-6, IL-10 and TGF-β, have been shown to impact negatively on islet function [35], with islet endocrine cells themselves secreting CCL2, CCL13, IL-6, IL-8, IL-1β and TNF-α, which contributes to a proinflammatory microenvironment [36].

Causality cannot be proven by analysis of associations in cross-sectional post-mortem tissue studies, underlying the need for dynamic functional studies to definitively elucidate the pathways hypothesised in this review.

## Potential role of profibrotic signalling in type 1 diabetes

Type 1 diabetes is characterised by T cell-mediated autoimmunity targeting the pancreatic beta cell [103, 104]. While classically considered to rapidly progress to absolute loss of all islet beta cells, it is now clear that some individuals with long-standing diabetes maintain residual beta cell mass and function [105, 106]. Two distinct histological endotypes have been proposed, with diagnosis at an older age associated with reduced intensity of islet autoimmunity and higher numbers of insulin-containing islets [107]. This suggests a more chronic inflammatory process with the potential for novel therapeutic approaches in parallel with current autoimmunity-targeted immunomodulatory strategies.

It has long been recognised that the pancreas is smaller in type 1 diabetes, with a loss of acinar volume and overall atrophy sparing only the uncinate lobe [108]. This is associated with reduced exocrine secretory capacity [109] and there has been much recent interest in unravelling the relationship between exocrine and endocrine pancreatic compartments in the initiation and progression of type 1 diabetes pathology, with reports increasingly focusing on inflammatory cells within the exocrine pancreas, including mononuclear, dendritic and T cells [110, 111]. Exocrine fibrosis is significantly increased in the type 1 diabetes pancreas, largely restricted to peri-ductal and peri-vascular regions [97, 112, 113]. It has previously been postulated that peri-ductal inflammation may play an initiating role in type 1 diabetes autoimmunity [114]. Further studies investigating the potential role of paracrine profibrotic signalling in type 1 diabetes onset and progression are underway. This includes the Hippo/YAP pathway believed to be a central driver of stellate cell activation and fibrosis in several tissues [115, 116]. YAP upregulation has recently been shown in pancreatic exocrine and endocrine compartments in type 1 diabetes autoantibody-positive individuals without diabetes and those with established type 1 diabetes [117].

Extracellular matrix changes in and around islets, characterised by hyaluronan deposition, have been reported in autoantibody-positive individuals and in established type 1 diabetes [118]. These changes are associated with the extent of lymphocytic insulitis and it may be that exocrine fibrosis is secondary to primarily islet-targeted autoimmunity in type 1 diabetes, possibly in association with acinar atrophy mediated by local insulin deficiency.

## Pivotal role for TGF-β signalling in driving the putative PSC/macrophage/islet perpetual cycle leading to fibrosis-associated diabetes

TGF-β is a key driver of fibrosis across a wide range of human tissues [119]. Within these tissues this cytokine directly activates resident fibroblasts inducing collagen synthesis and secretion and leading to extracellular matrix deposition and fibrosis [120]. In the pancreas these activated fibroblast phenotypes are characterised by α-SMA expression [121] and include PSCs and cancer-associated fibroblasts. Smooth muscle cells within larger blood vessels also express α-SMA and can be distinguished from activated fibroblasts by their location and by using additional specific markers [122]. α-SMA expression has also been shown in capillary pericytes [123]. Whether (peri)-islet pericytes play a role in the profibrotic signalling associated with beta cell dysfunction remains unclear, with further studies including additional markers (e.g. nerve/glial antigen 2 [NG2] for pericytes [123] and glial fibrillary acidic protein [GFAP] for PSCs [24, 121]) required. Epithelial tissue injury has been shown to be a key factor in initiating PSC activation in chronic pancreatitis [24, 36, 124–126]. TGF-β1 overexpression or direct administration in the murine pancreas has been shown to worsen or induce chronic pancreatitis, mirroring human pathology [127, 128]. As a secreted cytokine, immunostaining for TGF-β is challenging and studies have also evaluated cell-specific RNA expression by in situ hybridisation [29, 36, 129]. The highest level of *TGFB1* expression in mononuclear inflammatory cells was seen on single-cell RNA sequencing of samples obtained post pancreatectomy during islet autotransplant procedures (Y. Hang, Stanford Diabetes Research Centre, Stanford University School of Medicine, Stanford, CA, USA, personal communication).

PSCs have been shown to negatively impact endocrine cell viability and function in vitro and following intrapancreatic transplantation or co-transplantation with islets in in vivo models of type 2 diabetes [130–132]. Activation of macrophages and fibroblasts in parallel with epithelial cell stress-induced cytokine signalling in the presence of elevated TGF-β tissue levels have been implicated in hepatic fibrosis [133] and idiopathic pulmonary fibrosis (IPF) [134]. This supports our proposed perpetual cycle involving macrophages, PSCs and pancreatic endocrine cells themselves in the presence of elevated tissue TGF-β levels, leading to islet dysfunction (Fig. 3).

Administration of a TGF-β inhibitor in a mouse model of PDAC inhibited beta cell apoptosis [75]. Loss of beta cell end-differentiation evidenced by downregulation of urocortin-3 expression in a mouse model of type 2 diabetes was reversed by TGF-β inhibition [135]. Induction of proliferation in human beta cells has been shown following islet transplantation into a rodent model and administration of a small molecule TGF-β inhibitor [136].

In summary, PSC and macrophage activation in the presence of high tissue TGF-β levels leads to pancreatic fibrosis. This pathway may be an important mediator of fibrosis-associated pancreatic endocrine dysfunction reversible through TGF-β inhibition.

## Marketed therapeutic agents targeting TGF-β profibrotic signalling

Over the last 20 years, pirfenidone has become established as an antifibrotic therapy for IPF, reducing lung function decline [137, 138]. It is a synthetic pyridine that acts primarily through inhibition of TGF-β signalling through a range of mechanisms [139]. These include decreased TGF-β gene expression and biosynthesis together with reduced TGF-β-induced α-SMA expression, inhibiting PSC activation. Pirfenidone has been shown to reduce the severity of acute and chronic pancreatitis in mouse models in vivo and to induce quiescence in PSCs in vitro, reducing collagen deposition and the secretion of cytokines, including TNF-α and IL-6 [140–142]. Pirfenidone treatment reduced pancreatic macrophage number in a chronic pancreatitis mouse model, with inhibition of M1 polarisation on in vitro pirfenidone treatment [141]. Pirfenidone decreased PSC number and islet fibrosis in a rodent model of type 2 diabetes without a confirmed impact on beta cell mass or function [98].

Nintedanib is also licensed for IPF therapy and has been shown to reduce the rate of pulmonary function decline [143]. It is a tyrosine kinase receptor inhibitor with a potent effect on TGF-β signalling including inhibition of both tyrosine phosphorylation of the type II TGF-β receptor and Smad2/3 activation [144, 145]. Nintedanib has been shown to downregulate collagen production in activated fibroblasts [145, 146]. In a mouse model of chronic pancreatitis, in vivo administration of nintedanib led to decreased PSC activation, reduced collagen secretion and regression of fibrotic pathology [147].

Given the central role of TGF-β signalling in tissue homeostasis and repair [126] and the very high prevalence of pancreatic intraductal neoplastic (PanIN) lesions with the potential for malignant transformation [148, 149], it will be important to monitor recipients of pirfenidone and nintedanib for PDAC development. Accruing evidence, however, supports reduced carcinogenesis and progression following pirfenidone therapy, thought to be due to its positive impact on fibrosis and tumour-associated stroma. This includes decreased lung cancer incidence in individuals with IPF receiving pirfenidone therapy [150].

## Towards clinical trials of antifibrotic therapy targeting impaired beta cell function and diabetes

The majority of trials using existing antifibrotic therapies targeting TGF-β signalling have focused on non-pancreatic disease, with study endpoints specific to the primary target tissue (e.g. lung function in pulmonary fibrosis) [151]. A common challenge is a lack of biomarkers to accurately assess a primary impact on fibrosis. Non-invasive imaging modalities for assessing pancreatic fibrosis and response to treatment are currently being pioneered and include collagen-targeted PET imaging [152, 153].

Antifibrotic therapies may have additional potential for restoring endocrine functional mass in diabetes associated with PSC activation. The first randomised clinical trial using antifibrotic therapy in pancreatic disease is currently underway in the USA comparing pirfenidone with placebo in acute pancreatitis [154]. In this study, the investigators have included changes in inflammation- and fibrosis-related biomarkers as outcomes, for example CRP, TNF-α, IL-6, IL-8, IL-10 and angiopoietin-2. The study also includes diabetes endpoints including HbA_1c_ and fasting blood glucose levels at 6 months. There are to our knowledge no current trials evaluating antifibrotic therapy with the primary goal of improving beta cell function and glucose tolerance.

## Conclusion

Pancreatic fibrosis is a primary pathological feature in chronic pancreatitis, cystic fibrosis and PDAC and is also present in type 2 and type 1 diabetes. A vicious cycle initiated by and perpetuating epithelial damage involving ducts, PSCs and macrophages has been described in chronic pancreatitis, cystic fibrosis and PDAC. We propose a second cycle comprising PSCs, macrophages and paracrine signalling to and from ‘damaged’ islets as a potential common pathway leading to beta cell dysfunction and diabetes in all aetiologies associated with significant pancreatic fibrosis.

TGF-β is a key driver of fibrosis with therapeutics targeting this pathway in established clinical use for non-pancreatic indications and a first trial in pancreatitis underway. A strong case can be made for trials specifically exploring the efficacy of these agents in preventing and reversing fibrosis-associated diabetes. Such trials will require careful design to include measures of impact on pancreatic fibrosis and beta cell function/mass to test the potential for any truly disease-modifying impact, in parallel with validated person-reported outcome measures to assess positive and potentially negative impacts of therapies and overall impacts on well-being.

## Supplementary Information

Below is the link to the electronic supplementary material.

Slideset of figures (PPTX 1.80 MB)

## Abbreviations

α-SMA: Alpha-smooth muscle actin
CFRD: Cystic fibrosis-related diabetes
CFTR: Cystic fibrosis transmembrane conductance regulator
IPF: Idiopathic pulmonary fibrosis
PDAC: Pancreatic ductal adenocarcinoma
PDGF: Platelet-derived growth factor
PSC: Pancreatic stellate cell
