# Supplementary material for: The emerging role of pancreatic exocrine fibrosis as a common aetiological driver of islet dysfunction and diabetes: opportunities for novel disease-modifying interventions
Source: Diabetologia. 2026 Feb 10;69(5):1118–32. doi: 10.1007/s00125-026-06678-6 (PMC13005838; doi:10.1007/s00125-026-06678-6)
Supplement: Supplementary file 1 — Slideset of figures (PPTX 1.80 MB) [file 125_2026_6678_MOESM1_ESM.pptx]

## Slide 1
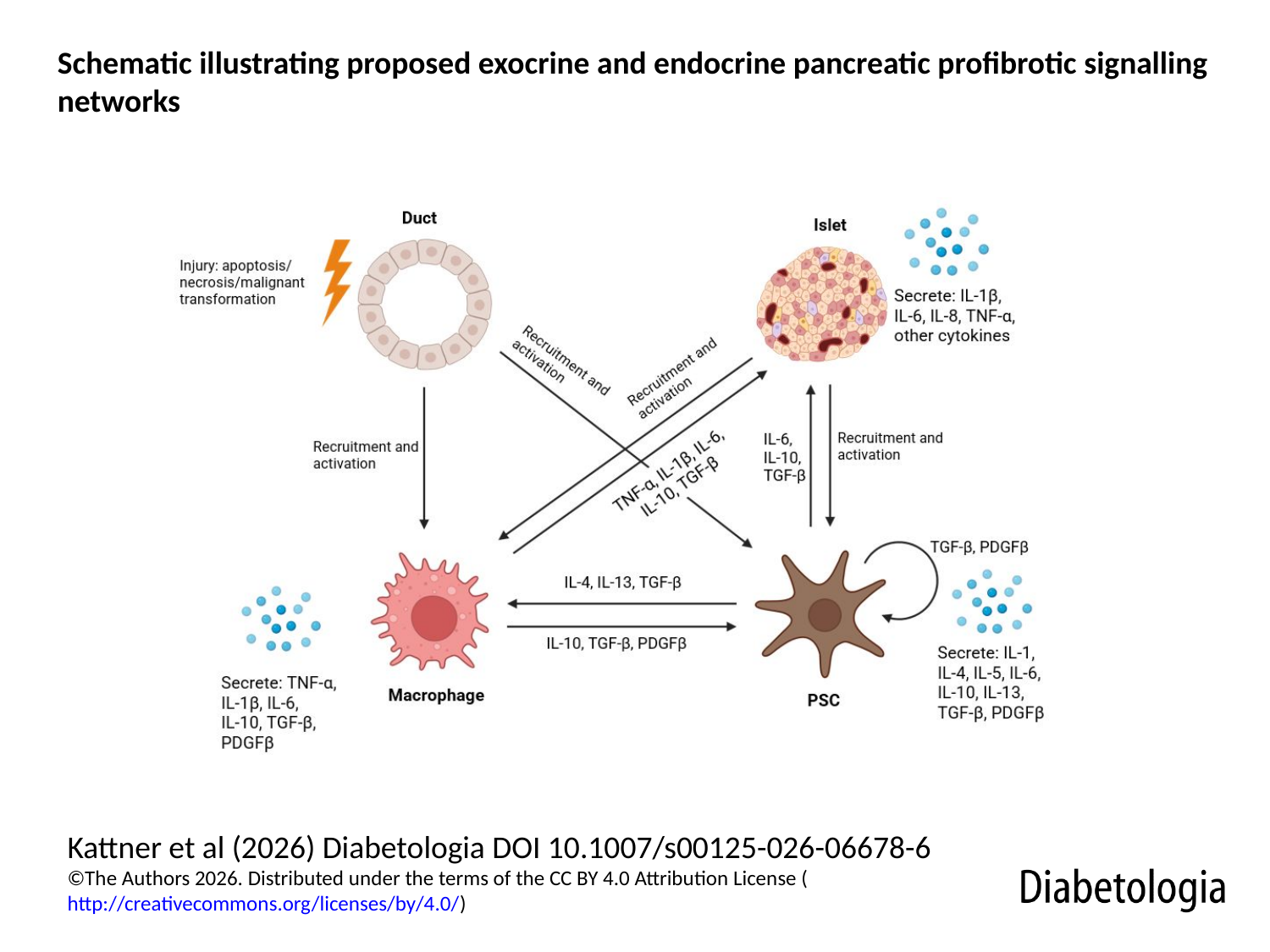

Schematic illustrating proposed exocrine and endocrine pancreatic profibrotic signalling networks
Kattner et al (2026) Diabetologia DOI 10.1007/s00125-026-06678-6
©The Authors 2026. Distributed under the terms of the CC BY 4.0 Attribution License (http://creativecommons.org/licenses/by/4.0/)

## Slide 2
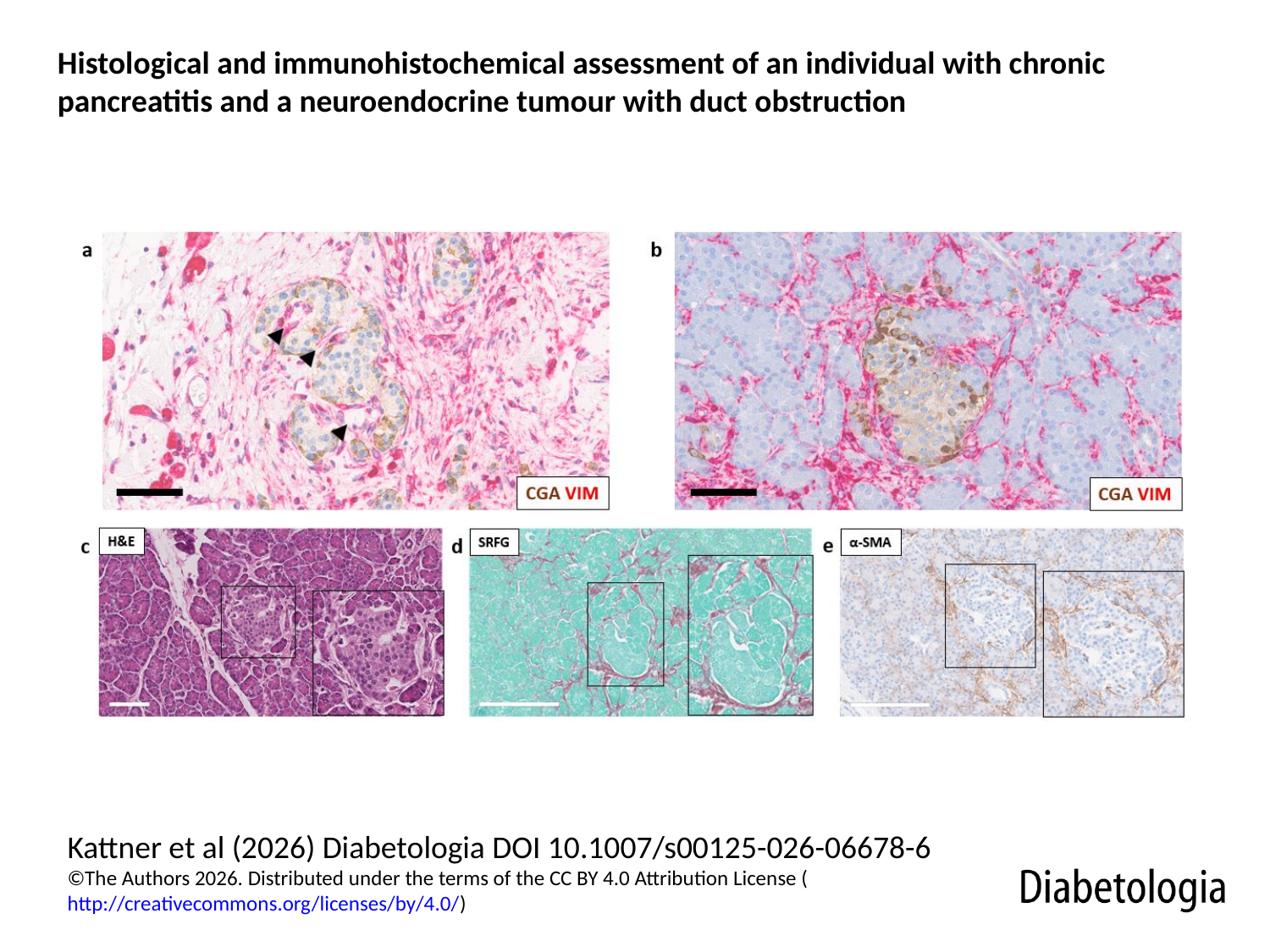

Histological and immunohistochemical assessment of an individual with chronic pancreatitis and a neuroendocrine tumour with duct obstruction
Kattner et al (2026) Diabetologia DOI 10.1007/s00125-026-06678-6
©The Authors 2026. Distributed under the terms of the CC BY 4.0 Attribution License (http://creativecommons.org/licenses/by/4.0/)

## Slide 3
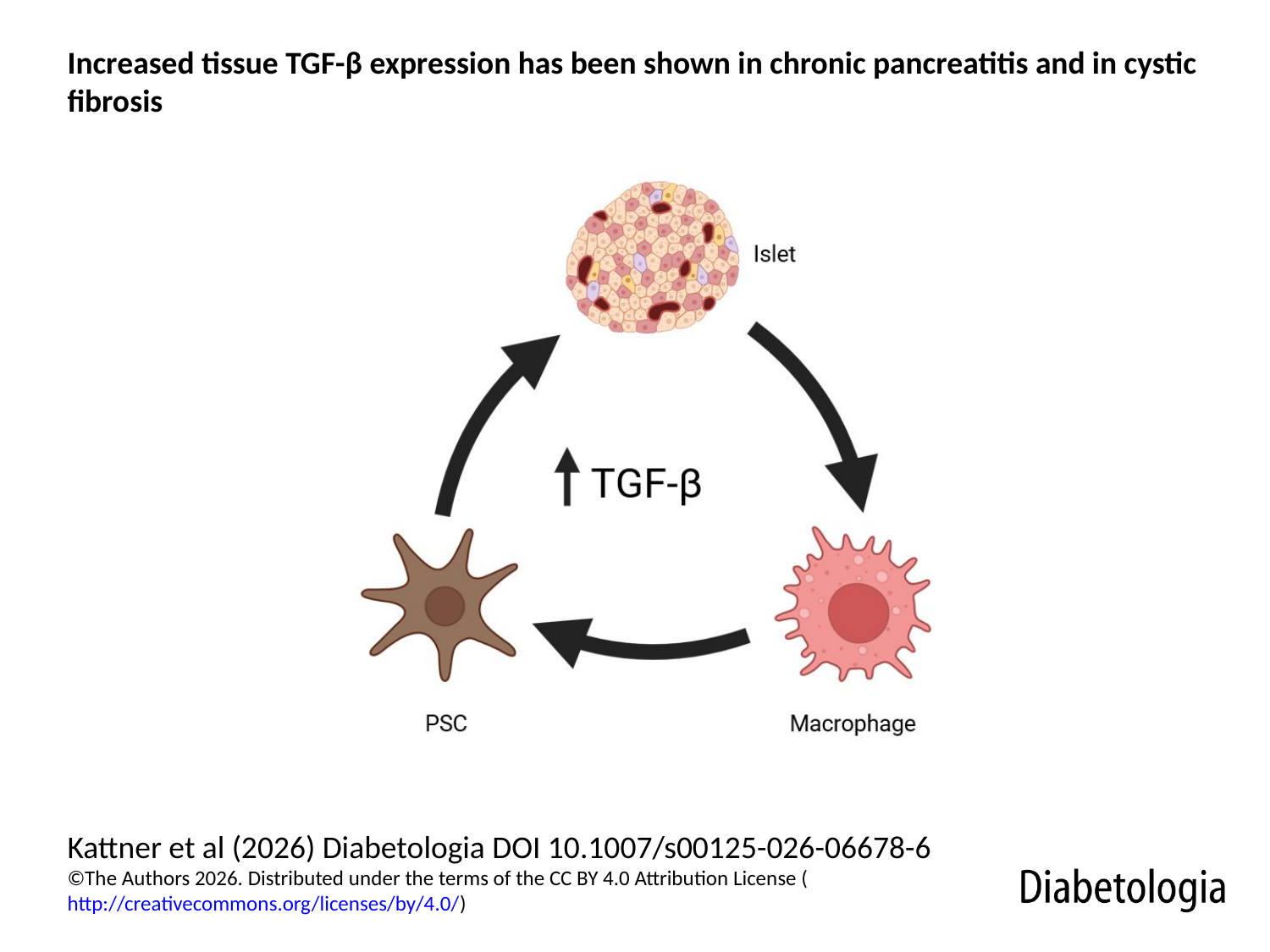

Increased tissue TGF-β expression has been shown in chronic pancreatitis and in cystic fibrosis
Kattner et al (2026) Diabetologia DOI 10.1007/s00125-026-06678-6
©The Authors 2026. Distributed under the terms of the CC BY 4.0 Attribution License (http://creativecommons.org/licenses/by/4.0/)

## Slide 4
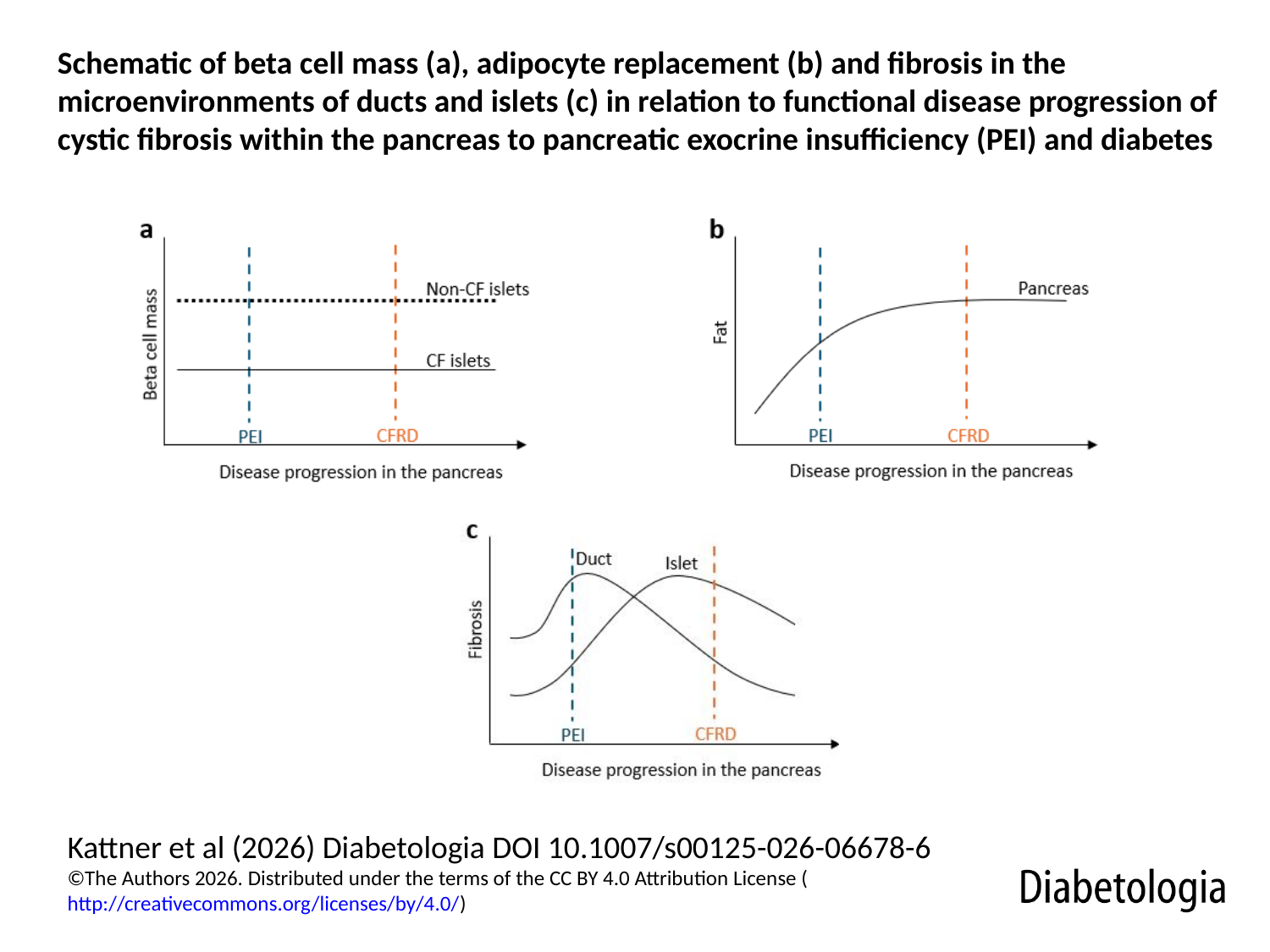

Schematic of beta cell mass (a), adipocyte replacement (b) and fibrosis in the microenvironments of ducts and islets (c) in relation to functional disease progression of cystic fibrosis within the pancreas to pancreatic exocrine insufficiency (PEI) and diabetes
Kattner et al (2026) Diabetologia DOI 10.1007/s00125-026-06678-6
©The Authors 2026. Distributed under the terms of the CC BY 4.0 Attribution License (http://creativecommons.org/licenses/by/4.0/)
